# Educative accompaniment. Scoping review of academic publications

**DOI:** 10.3389/fmed.2025.1668756

**Published:** 2025-11-25

**Authors:** Jorge López González, Paula Crespí, María Fernanda Gambarini

**Affiliations:** Faculty of Education and Psychology, Accompaniment Institute, Universidad Francisco de Vitoria, Madrid, Spain

**Keywords:** educational accompaniment, educative accompaniment, scoping review, integral formation, accompaniment, theoretical conceptions, academic publications

## Abstract

**Introduction:**

Educational accompaniment (EA) has gained increasing relevance in recent years, particularly in Spain, with applications across education, healthcare, and social domains. Despite its growing presence, there is no comprehensive synthesis of the academic literature on EA. This review aims to identify and analyze the main models, definitions, and benefits associated with EA.

**Methods:**

A scoping review was conducted using three databases: Web of Science, Scopus, and nDICEs-CSIC. The search focused exclusively on the term educational accompaniment, which narrowed the scope but ensured conceptual specificity. Inclusion criteria targeted studies that explicitly addressed EA, excluding those using the term in a colloquial or unrelated sense. A total of 28 studies were selected and analyzed following PRISMA guidelines.

**Results:**

The review identified four distinct paradigms of EA: psychosocial, personalist, socio-educational, and competency-based. Each model is grounded in specific theoretical and methodological frameworks. An integrated definition of EA is proposed as an intentional educational action through which one person enlightens and supports another in their integral development, facilitating their autonomy, maturity, and realization of a fulfilling life. Key findings highlight the importance of the quality of the accompaniment relationship, sustained engagement, practitioner training, and stakeholder coordination. Reported benefits include enhanced learner autonomy, holistic development, and improved employability.

**Discussion:**

EA emerges as a salient and evolving discipline with strong theoretical foundations and practical relevance. It is increasingly recognized as a valuable pedagogical approach in diverse fields, including education, healthcare, and social services, where its relational and formative nature supports autonomy, personal growth, and holistic development.

## 1 Introduction

The use of the term accompaniment has been gaining interest among sociologists, psychologists, and health professionals, among others (1–3). Educational accompaniment (EA) has also developed significantly, especially in the university context, with various modalities and forms of application (4–6). Previous reviews of educational accompaniment indicate the relevance of the topic, both theoretically and empirically, as well as the importance of conducting a scoping review to “advance the definition of a well-articulated theoretical conceptual framework and obtain evidence on educational outcomes” [(7), p. 234].

Accordingly, this article presents the conceptualizations, typologies, principal stakeholders, domains of application, and evidenced benefits of EA, as revealed through our scoping review. The search conducted focuses exclusively on the use of the term educational accompaniment, which limits the results obtained, although it constitutes a starting point for future research that will make it possible to relate the different concepts and types of EA even when this term is not used.

Etymologically, “accompaniment” denotes companionship—literally, the sharing of one’s bread with a fellow traveler (8–10). In Biblical Hebrew and Greek, the term for companion signifies one who “is with me,” implying mutual support (cf. Gn 2:20). The field of medicine—and healthcare more broadly—exemplifies this principle: healers are called to draw near to the sick, walk alongside them, and share their resources in solidarity (11). Likewise, educational accompaniment traces its roots to ancient pedagogical traditions: Pedagogy (from the Greek *paideia)* etymologically refers to someone who accompanies and guides students (12).

Educational accompaniment has undergone significant development in recent years, with applications across multiple domains, psychological, social, healthcare, educational, pedagogical, and spiritual, among others (13–16). Educational accompaniment has been implemented in formal education settings (schools and universities, including healthcare practitioners), in social education, and in the training of future teachers, healthcare professionals or physicians (17). Álvarez-Montero et al. (18) highlight the importance of educative accompaniment in promoting the integral development of medical students, especially in emotionally complex areas such as coping with death and grief.

The concept of educational accompaniment employed is closely related to that of personalized care and education (9, 19). Personalized education may be regarded as a broader construct than accompaniment. To personalize is not merely to adapt the educational process to the learner’s specific characteristics but to conduct it in collaboration with the individual (20), thereby contributing to their intellectual and personal perfection. Personalized education can be applied to different functions, not only to accompaniment, but also to instruction and assessment (or diagnosis), for example. However, it is in accompaniment where personalized education is most evident. Personalized education is an educational theory focused on learning processes and curriculum content. Accompaniment, on the other hand, is an activity, a relational action that may or may not be linked to personalized education. As will be seen later, there are various models or conceptualizations of accompaniment underpinned by different educational theories. In any case, accompaniment transcends the curricular level, focusing on the development of maturity, autonomy, and the individual’s sense of purpose in life.

Educational accompaniment (EA) is also employed as a pedagogical strategy intended to facilitate the teaching–learning process—whether for children, vulnerable adults, or healthcare workers. Additional objectives associated with EA include the full development and formation of the learner, as well as the support of autonomy and emancipation for individuals at risk of social exclusion or with disabilities.

The scoping review methodology does not aim to generate new empirical data but rather to appraise the information provided by the most relevant studies on the topic (21, 22). A scoping review enables a systematic synthesis of knowledge by identifying key concepts, theories, sources, and evidence (23).

The specific objectives (O) of this research on EA are as follows: (O1) to chart the temporal evolution of EA publications and their distribution by journal, author, and country; (O2) to analyze the principal definitions, theoretical models, and founding authors; (O3) to identify the research methodologies employed; (O4) to determine the types or modalities of accompaniment; (O5) to observe the domains in which it is applied; (O6) to identify who acts as accompanier and who as accompanied; (O7) to analyze the benefits; (O8) to highlight the difficulties; (O9) to determine the key elements; (O10) to identify the core competencies; (O11) to identify the principal research findings; (O12) to offer a forward-looking perspective on educational accompaniment.

## 2 Methodology

### 2.1 Search strategy and eligibility criteria

As previously noted, the scoping review methodology aims to map and organize the existing scientific literature on a given topic, rather than to conduct a systematic review assessing the effectiveness of the studies (24). The scoping review approach adopted in this study follows the PRISMA guidelines (*Preferred Reporting Items for Systematic Reviews and Meta-Analyzes*) (25).

To conduct the review, three databases were consulted: (1) WOS (Web of Science), (2) SCOPUS, and (3) ÍnDICEs-CSIC. The first two provide international coverage, whereas ÍnDICEs-CSIC is a multidisciplinary bibliographic repository maintained by the Spanish National Research Council, primarily indexing and disseminating research articles from Spanish scientific journals.

The search terms were: “educative accompaniment” OR “educational accompaniment” OR “accompaniment educational.” This search is therefore based on the use of the term in literature, not on the use of the concept itself. It has the limitation that it omits some contributions that address accompaniment under other names (mentoring, for example) because the term does not appear in the publication. However, it has the advantage of allowing the research to be better narrowed down to works that treat accompaniment as a category in its own right. As we shall see, the term accompaniment has characteristics that justify its differentiated use from related but more specific terms such as mentoring or coaching.

The exclusion criteria used are: (1) duplicate records are eliminated; (2) works that used the term “accompaniment” in a colloquial sense, simply as “being with others” or paraphrasing “follow-up” are eliminated; (3) works that do not deal specifically with educational accompaniment, but rather with other issues (e.g., spiritual accompaniment) are eliminated; (4) works written in a language other than Spanish or English are eliminated.

The search was carried out without restriction on the initial publication date (from the beginning of the database) until 9 September 2023.

### 2.2 Data collection

For each selected study, the following descriptive data were extracted: (1) year of publication; (2) authors; (3) country of publication; (4) title; (5) journal or book name; (6) definition of accompaniment; (7) foundational authors; (8) theoretical model employed; (9) research methodology; (10) type of educational accompaniment; (11) research findings; (12) prospective outlook; (13) educational setting; (14) area or discipline; (15) accompanier; (16) accompanied individual; (17) benefits of accompaniment; (18) challenges to accompaniment; (19) key elements of accompaniment; (20) key competencies of accompaniment.

## 3 Results

### 3.1 Selected studies

**FIGURE 1:**
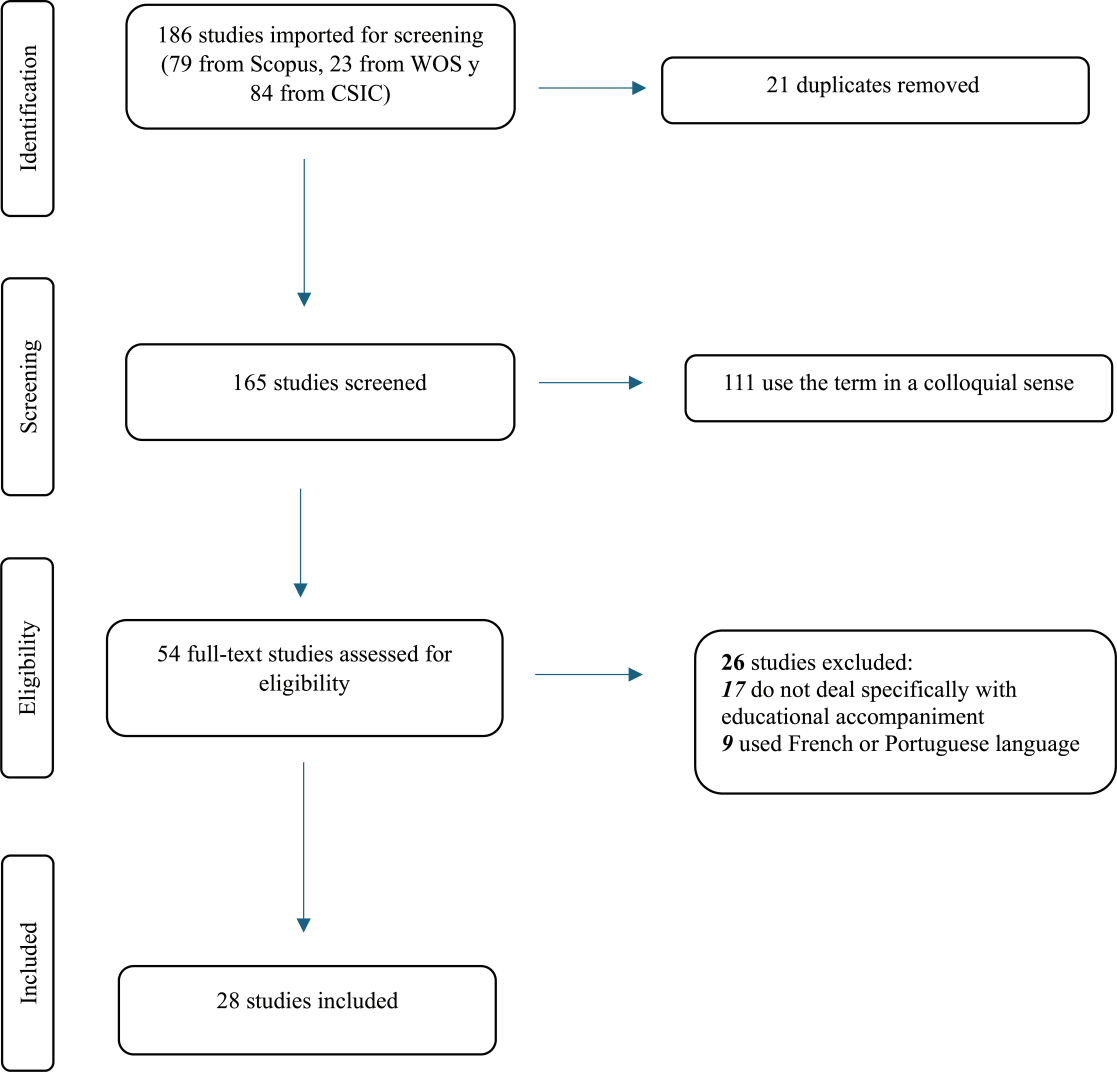
PRISMA diagram flow of study selection.

The PRISMA flow diagram (Figure 1) illustrates the selection process for the 28 EA publications included in the scoping review.

Table 1 presents the 28 selected studies, along with the numerical reference assigned to each study in this article.

**TABLE 1:** Characteristics of educational accompaniment (EA) models.

No.	Studies	Title
1	Pallarès Piquer et al. (26)	Pedagogía del cuerpo y acompañamiento, una combinación al servicio de los retos de la educación.
2	Planella Ribera (27)	Pedagogía Social y diversidad funcional: de la rehabilitación al acompañamiento.
3	Rodríguez Herrero et al. (28)	Orientaciones pedagógicas para el acompañamiento educativo por duelo a personas adultas con discapacidad intelectual.
4	Vélaz de Medrano Ureta (29)	Competencias del profesor-mentor para el acompañamiento al profesorado principiante.
5	Arnaus i Morral (30)	La relación como práctica política en la formación inicial de educadoras y educadores sociales.
6	Pàmies Rovira et al. (31)	(Des)vinculación escolar y procesos de acompañamiento en educación secundaria por clase y origen en un municipio de la región metropolitana de Barcelona.
7	Cárdenas Puyo and Tovar-Gálvez (32)	Computadores y red en Colombia: posibilidades de interacción globalizadora en instituciones educativas públicas y desarrollo regional.
8	Arancibia Muñoz et al. (33)	Valoración y barreras en la integración del e-portafolio en el proceso de práctica inicial por parte de docentes y estudiantes de Educación Superior.
9	García Gràcia and Sánchez-Gelabert (34)	La heterogeneidad del abandono educativo en las transiciones posobligatorias. Itinerarios y subjetividad de la experiencia escolar.
10	Montserrat and Melendro (35)	>Qué habilidades y competencias se valoran de los profesionales que trabajan con adolescencia en riesgo de exclusión social? Análisis desde la acción socioeducativa.
11	Martínez Clares et al. (36)	Una (re)visión de la tutoría universitaria: la percepción de estudiantes y tutores de estudios de Grado
12	Aravena-Kenigs et al. (37)	Incidence of the Pedagogical Accompaniment with Use of Rubrics in the Improvement of the Teaching Performance.
13	Boroel Cervantes et al. (38)	Educación exitosa para todos: la tutoría como proceso de acompañamiento escolar desde la mirada de la equidad educativa.
14	Herrera-Pastor et al. (39)	Acompañamiento socioeducativo y resiliencia en jóvenes migrantes extutelados: Una aproximación desde las oportunidades, los sueños y los apoyos.
15	Álvarez-Gallego et al. (40)	Estrategias de Acompañamiento Educativo y Familiar en la Educación Inicial: una revisión teórica.
16	García Lara et al. (41)	Los padres como maestros: campo de tensión en escenarios rurales y urbanos de Chiapas, México.
17	Fernández-Simo and Cid Fernández (42)	Análisis longitudinal de la transición a la vida adulta de las personas segregadas del sistema de protección a la infancia y a la adolescencia.
18	Fernández-Simo et al. (43)	Buenas prácticas y oportunidades de mejora en el acompañamiento socioeducativo con juventud en protección durante la transición a la vida adulta.
19	Crespí and López (44)	Mentoring impact on the transversal competence’s development. An experience of educational accompaniment in the integral formation of the university student.
20	Viñado Oteo and Miró López (45)	Gestar una cultura de acompañamiento como clave para la formación en la universidad desde la mirada de Benedicto XVI.
21	Salinas Tomás (46)	El modelo de acompañamiento en las empresas de inserción y su incidencia en la empleabilidad de personas en situación y/o riesgo de exclusión social.
22	Sevillano-Monje and Martín-Gutiérrez (47)	Programa de apoyo a la transición a la vida adulta en Andalucía para la juventud extutelada. Potencialidades y retos en los márgenes de la emancipación.
23	Herrera-Pastor et al. (48)	Acompañamiento sociopedagógico, holismo y longitudinalidad: Claves de una buena práctica con un menor infractor.
24	González-Iglesias and De la Calle Maldonado (49)	El acompañamiento educativo, una mirada ampliada desde la antropología personalista.
25	Sevillano-Monje and Sanz-Escutia (50)	Percepciones de los profesionales sobre el acompañamiento socioeducativo en los recursos de transición a la vida adulta. Un análisis comparativo entre Andalucía y Cataluña.
26	Jiménez-Jiménez et al. (51)	El espacio como elemento didáctico en los procesos de acompañamiento socioeducativo con adolescentes.
27	Blanchard and Fernandes Procópio (52)	Claves y proceso para configurar la identidad del docente educador desde la formación inicial.
28	Castro Pérez and Moya Márquez (53)	El acompañamiento pedagógico en la práctica profesional presencial y/o virtual.

The 28 studies included in this scoping review are identified in the reference list with an asterisk (*).

### 3.2 Main results

The following results are presented in alignment with the specific objectives outlined above.

#### 3.2.1 Temporal evolution and localization of the research (O1)

Table 2 provides the general data on the volume and temporal evolution of the scientific literature on EA.

**TABLE 2:** Evolution of the number of publications on educational accompaniment (EA).

No.	3	1	4	5	6	9
Date	< 2010	2010–12	2013–15	2016–18	2019–21	2022–23

An increase in the number of publications on EA is evident, particularly during the 2022–23 period. With respect to authorship, the vast majority have contributed a single study to this selection. Four authors have authored two publications each (Fernández-Simo, from Universidad de Vigo; Sevillano-Monje, from Universidad de Extremadura; Cid Fernández, from Universidad de Vigo and Herrera-Pastor, from Universidad de Málaga). A single author, Planella Ribera, affiliated with the Universitat Oberta de Catalunya, has three publications.

The majority of journals included in this review have featured only one article on EA and are based in Spain or Ibero-America countries. Four journals have published two articles apiece: *Teoría de la Educación*, *Revista de Medios y Educación*, *Scientia et Fides*, and *Pedagogía Social*. These are journals linked to the Spanish university sector. In terms of geographic focus, research on EA is dominated by Spain (22 publications), followed by Colombia, Chile, and Mexico (two publications each).

#### 3.2.2 Definitions, theoretical models, and foundational authors (O2)

A review of the included studies reveals that 13 do not provide a formal definition of the term accompaniment but rather offer a generic approximation such as “providing support” to another person. The notion of accompaniment is frequently conveyed via the “conceptual metaphor” of a journey (54), whereby accompaniment is understood as “walking alongside someone” [(27), p. 124], “being accessible and available at the side of a person in order to traverse a path together” [(51), p. 62], or engaging in a “harmonized path of encounters and misencounters” [(49), p. 189]. While these expressions are not strict definitions, they carry significant epistemological value.

Four definitions stand out for their precision in explicating the concept of accompaniment, its nature, characteristics, and purpose: (1) Planella Ribera [(27), p. 124] describes educational accompaniment as “integrating all initiatives, methods, and practices aimed at empowering individuals with support needs to proceed independently.” (2) Boroel Cervantes et al. (38), p. 96, drawing on Puerta Gil (55), define educational accompaniment as “an intentional educational action grounded in closeness and a willingness to influence and be influenced by the other, with the overarching horizon of potentiating their capacities so that they may continue to shape and mold their dreams.” (3) Crespí and López [(44), p. 2] characterize educational accompaniment as “an intentional pedagogical action that aims to help and support people in their effort to know themselves and take decisions that favors their personal growth and development, with the necessary support in its implementation.” (4) Salinas Tomás [(46), p. 13] refers to educational accompaniment as the “pedagogical relationship whose primary content is the attainment of personal autonomy, independence, or emancipation in three dimensions: technical-professional, socio-occupational, and personal.” This definition is drawn from a manual produced by the Federation of Insertion Enterprises Associations [(56), p. 72].

Despite their differences, these definitions share several core elements regarding EA: (1) nature: EA is an intentional methodology or educational action; (2) purpose: EA seeks to enable the accompanied individual to develop their capacities or competencies to achieve a full life characterized by maturity, autonomy, or virtue; (3) distinctive feature: the accompanier illuminates and sustains the accompanied in a non-directive manner, allowing free decision-making.

No author has been identified as appearing repeatedly in the review of the literature on accompaniment. Nonetheless, certain foundational authors emerge as foundational of distinct schools of thought that continue to inform accompaniment research. Although each EA model tends to align more closely with particular theorists, many of these figures exert influence across multiple paradigms. For example, Rogers (57, 58), in *Counseling and Psychotherapy*, articulates a non-directive therapeutic approach that is central to the psychosocial model yet also resonates within other EA frameworks.

Overall, the various research articles included in our scoping review can be organized into four theoretical models: (1) the psychosocial model, (2) the personalist model, (3) the socio-educational model, and (4) the competency-based model. Twenty-five of the 28 studies align with one of these four frameworks, each assigned to the model that best corresponds with its thematic development. The remaining three studies (nos. 7, 8, and 9) could not be categorized due to insufficient or unclear theoretical elaboration.

Table 3 provides a concise overview of the key characteristics of these four theoretical models of EA.

**TABLE 3:** Characteristics of educational accompaniment (EA) models.

Model	Psychosocial	Personalist	Socio-educational	Competency-based
No. of studies (%)	5 (17.9%)	6 (21.4%)	8 (28.6%)	6 (21.4%)
Studies	1, 2, 3, 6, and 10	5, 16, 19, 20, 24, and 27	14, 15, 17, 18, 21, 22, 23, and 25	4, 11, 12, 13, 26, and 28
Concept	A non-directive methodology for working with learners aimed at their personal improvement (health and maturity)	A dialogical educational action oriented toward the unfolding of relationships and capacities for the person’s fullness	An educational relationship aimed at emancipation and integration into the socio-occupational and professional sphere of at-risk individuals	An educational action grounded in relational skills, oriented toward the excellent exercise of the individual’s capacities
Foundational authors	Rogers (57, 58)	Mounier (59)	Freire (60)	Boyatzis (61)
Emphasis	Relational empathy	Relationships/community	Social adaptation	Competency development
Threshold concept	Non-directivity	Encounter	Autonomy	Competencies
Purpose	Health	Fulfilment of life	Social integration and quality of life	Excellence

#### 3.2.3 Reserach methodology (O3)

Table 4 presents the findings concerning the research methodologies employed in the analyzed studies. As shown, the most frequently used methodology is qualitative (46.4%), followed by theoretical essay (32.1%) and mixed methods (21.4%).

**TABLE 4:** Research Methodologies in educational accompaniment (EA).

Methodology	Qualitative	Theoretical study	Mixed (quantitative–qualitative)
No. of studies (%)	13 (46.4%)	9 (32.1%)	6 (21.4%)
Studies	7, 8, 13, 14, 15, 16, 17, 18, 22, 23, 25, 26, and 28	1, 2, 3, 4, 5, 20, 21, 24, and 27	6, 9, 10, 11, 12, and 19
Typology	Phenomenological design using focus groups, semi-structured and in-depth interviews, SWOT analysis, analytic journals, longitudinal study, participant observation in case studies, and participatory action research	In-depth exercise of discernment and synthesis	Combination of quantitative and qualitative methods aimed at achieving a broader and more comprehensive approach
Reference authors	Boroel Cervantes et al. (38), Herrera-Pastor et al. (39), Álvarez-Gallego et al. (40), García Lara et al. (41), Fernández-Simo et al. (43), Castro Pérez and Moya Márquez (53)	Pallarès Piquer et al. (26), Vélaz de Medrano Ureta (29), Salinas Tomás (46), González-Iglesias and De la Calle Maldonado (49), Blanchard and Fernandes Procópio (52)	Pàmies Rovira et al. (31), García Gràcia and Sánchez-Gelabert (34), Aravena-Kenigs et al. (37), Crespí and López (44)

#### 3.2.4 Types of accompaniment (O4)

Table 5 presents the results related to the typology of accompaniment addressed by the analyzed studies.

**TABLE 5:** Types of accompaniment.

Typology	Tutoring	Mentoring	Pedagogical guidance	Informal encounters
No. of studies (%)	5 (17.9%)	2 (7.1%)	2 (7.1%)	2 (7.1%)
Studies	3, 13, 18, 25, 27	4, 19	12, 28	18, 26
Concept	Support that promotes holistic development; learning and competency development; and a more humane approach to teaching	Guidance that facilitates development in any domain by fostering transversal competencies	Support that enhances the teaching–learning process	Support provided through spontaneous conversations and in everyday or recreational activities
Reference authors	Rodríguez Herrero et al. (28), Boroel Cervantes et al. (38), Sevillano-Monje and Sanz-Escutia (50), Blanchard and Fernandes Procópio (52)	Vélaz de Medrano Ureta (29), Crespí and López (44)	Aravena-Kenigs et al. (37), Castro Pérez and Moya Márquez (53)	Fernández-Simo et al. (43), Jiménez-Jiménez et al. (51)

As shown in Table 5, the majority of studies address tutoring (17.9%), which is implemented across a range of contexts—including school education, social education, and teacher training. While tutoring is most common in the school setting, its significance in special education is also recognized (28). In higher education, the tutor is portrayed as an experienced instructor who guides the future teacher through their initial professional experiences (52).

A mentor is characterized as an individual possessing experience and maturity, who provides guidance, advice, or reinforcement to support the mentee’s integral development and the acquisition of transferable competencies applicable to personal, social, and professional domains (29, 44).

Pedagogical guidance functions as a specialized form of tutoring, wherein an advising teacher offers formative support to a colleague, enabling the exchange of experiences and the enhancement of the teaching–learning process (37, 53)

Finally, the category of informal encounters encompasses: (1) spontaneous and unscheduled conversations and meetings; (2) informal accompaniment during everyday activities (43); and (3) dialogic, recreational, and technical spaces for interaction (51).

#### 3.2.5 Educational context (O5)

Table 6 displays the educational context in which EA is implemented.

**TABLE 6:** Educational context of educational accompaniment (EA).

Educational context	Social education	Higher education	School education	Special education
No. of studies (%)	9 (32.1%)	8 (28.6%)	8 (28.6%)	2 (7.1%)
Studies	6, 14, 17, 18, 21, 22, 23, 25, and 26	5, 8, 11, 19, 20, 24, 27, and 28	4, 6, 7, 9, 12, 13, 15, and 16	2 and 3

#### 3.2.6 Roles of the accompanier and the accompanied (O6)

The term *accompanier* denotes an educator who may be “either the teacher or anyone contributing to the educational endeavor” [(49), p. 186]. A *mentor* is understood to be an individual possessing profound life maturity and wisdom who guides the *apprentice* on their path to development, excellence, and fullness (44, 45, 62). As Crespí and López [(44), p. 3] state, the accompanier “must be an educator with demonstrable maturity and experience in educational accompaniment” [(44), p 3]. The predominant accompanier roles identified are teachers (50%), followed by social educators (21.4%), with the remaining roles (see Table 7) less frequently represented.

**TABLE 7:** Roles of the accompanier and the accompanied.

Accompanier	No. of studies (%)	Studies
Teacher	14 (50%)	4, 5, 6, 9, 11, 12, 13, 15, 20, 24, 25, 26, 27, and 28
Social educator	6 (21.4%)	2, 3, 10, 14, 17, 18
Social worker	2 (7.1%)	21 and 23
Parents	2 (7.1%)	5 and 16
School administrator	1 (3.6%)	12
Guidance counselor	1 (3.6%)	22
EA professional	1 (3.6%)	19
Mediator	1 (3.6%)	3
Student (as facilitator)	1 (3.6%)	14
**Accompanied**	**No. of studies (%)**	**Studies**
Vulnerable individuals	8 (28.6)	10, 14, 17, 18, 21, 22, 23, and 25
University students	7 (25%)	5, 11, 19, 20, 24, 27, and 28
School students	5 (17.9%)	6, 9, 13, 15, and 26
Teachers	4 (14.3%)	4, 7, 8, and 12
Persons with disability	2 (7.1%)	2 and 3
School-aged children	1 (3.6%)	16

The *accompanied individual* is broadly conceived as a learner who seeks “to discover their calling and to respond freely to it” [(49), p. 188]. This role encompasses apprentices or students “with vital questions, concerns, longings, and fears that need to be awakened, explored, and initially addressed” [(44), p. 3]. Among the profiles identified, students, whether at the secondary or tertiary level, constitute the largest group (42.9%), followed by individuals in vulnerable circumstances (28.6%) and educators (14.3%), with the remaining roles represented to a lesser extent. The remaining roles (Table 7) are less represented.

#### 3.2.7 Benefits of accompaniment (O7)

Table 8 summarizes the benefits of educational accompaniment identified in this review. The most frequently reported benefit is enhancement of autonomy (39.3%), followed by social inclusion (25.0%), integral formation (21.4%), employability (17.9%), and development of transversal competencies (17.9%).

**TABLE 8:** Benefits of educational accompaniment (EA).

Benefit	No. of studies (%)	Studies	Associated improvements	Reference authors
Autonomy	11 (39.3%)	1, 2, 10, 14, 17, 18, 21, 22, 23, 25, and 26	Facilitates independence or emancipation in educational, personal, social, and occupational domains.	Herrera-Pastor et al. (39), Fernández-Simo and Cid Fernández (42)
Social inclusion	7 (25%)	14, 17, 18, 21, 22, 23, and 25	Promotes social adaptation, relationship building, and the formation of stable, trusted support networks	Fernández-Simo and Cid Fernández (42), Fernández-Simo et al. (43), Herrera-Pastor et al. (48)
Integral formation	6 (21.4%)	11, 15, 19, 20, 24, and 27	Develops all dimensions of the individual: cognitive, affective, communicative, ethical, social, bodily, and spiritual	Crespí and López (44), Viñado Oteo and Miró López (45)
Employability	5 (17.9%)	4, 21, 22, 25, and 28	Facilitates entry into the workforce or professional practice	Vélaz de Medrano Ureta (29), Salinas Tomás (46), Castro Pérez and Moya Márquez (53)
Transversal competencies	5 (17.9%)	4, 13, 19, 26, 27	Cultivates transversal competencies transferable across personal, social, and professional context	Boroel Cervantes et al. (38), Crespí and López (44), Jiménez-Jiménez et al. (51), Blanchard and Fernandes Procópio (52)
Learning achievement	3 (10.7%)	13, 15, and 16	Enhances the teaching–learning process and achievement of learning outcomes	Boroel Cervantes et al. (38), García Lara et al. (41)
School engagement	2 (7.1%)	6 and 9	Strengthens attachment to educational settings and reduces school dropout	Pàmies Rovira et al. (31), García Gràcia and Sánchez-Gelabert (34)
Teaching performance	2 (7.1%)	5 and 12	Supports professional development and strengthens instructional practice	Arnaus i Morral (30), Aravena-Kenigs et al. (37)
Digital competence	2 (7.1%)	7 and 8	Promotes the use of Information and Communication Technologies (ICT) in the teaching-learning process	Cárdenas Puyo and Tovar-Gálvez (32), Arancibia Muñoz et al. (33)
Mental health	1 (3.6%)	3	Supports mental health among adults with intellectual disabilities in grief situations	Rodríguez Herrero et al. (28)

#### 3.2.8 Difficulties to effective accompaniment (O8)

Table 9 presents the difficulties or challenges encountered in implementing high-quality educational accompaniment.

**TABLE 9:** Challenges to effective educational accompaniment (EA).

Challenge diversity	Diversity of needs	Unified direction	Continuity	Motivation
Number of studies (%)	7 (25%)	4 (16%)	3 (10.7%)	1 (3.5%)
Studies	2, 3, 6, 13, 17, 23, 28	11, 12, 15, 16	18, 22, 25	8
Explanation	The educational needs of the accompanied individual are highly diverse and require exceptionally qualified professionals	A cohesive leadership and a shared pedagogical vision are required	Ongoing follow-up over time, continuity across transitional stages, and sustained institutional support are necessary	Sustained motivation, particularly on the part of the accompanier, is essential
Reference authors	Rodríguez Herrero et al. (28), Boroel Cervantes et al. (38), Castro Pérez and Moya Márquez (53)	Aravena-Kenigs et al. (37), Álvarez-Gallego et al. (40), García Lara et al. (41)	Pàmies Rovira et al. (31), Fernández-Simo et al. (43), Sevillano-Monje and Martín-Gutiérrez (47)	Arancibia Muñoz et al. (33)

The heterogeneity and complexity of the accompanied individuals’ educational needs pose a significant challenge (38), as does the cognitive vulnerability of certain learners (28). Moreover, the absence of a shared pedagogical vision among leadership structures (37) and misalignment with the expectations and demands of both the accompanied individual and their family further complicate effective implementation of EA (40, 41).

#### 3.2.9 Key elements/conditions for effective accompaniment (O9)

Table 10 outlines the critical elements necessary for effective EA.

**TABLE 10:** Key elements for effective educational accompaniment (EA).

Key factor	Interpersonal relationship	Planning and space organization	Accompanier training	Practical experience
No. of studies (%)	22 (78.6%)	5 (17.9%)	4 (14.3%)	1 (3.6%)
Studies	1, 3, 4, 5, 10, 11, 12, 13, 14, 15, 17, 18, 19, 20, 21, 22, 23, 24, 25, 26, 27, and 28	11, 18, 19, 26, and 28	1, 3, 4, and 19	19
Explanation	A relational dynamic characterized by trust and personalized attention	Organizational considerations related to timing and physical/virtual spaces	The maturity and personal competencies of the accompanier	The accompanier’s prior experience
Reference authors	Aravena-Kenigs et al. (37), Herrera-Pastor et al. (39), Fernández-Simo and Cid Fernández (42), Fernández-Simo et al. (43), Crespí and López (44)	Crespí and López (44), Jiménez-Jiménez et al. (51)	Pallarès Piquer et al. (26), Rodríguez Herrero et al. (28), Vélaz de Medrano Ureta (29), Crespí and López (44)	Crespí and López (44)

A salient finding across all studies is the centrality of the relational factor in effective accompaniment. Scholars consistently identify the interpersonal dynamics between accompanier and learner as the pivotal element of EA, noting that a trusting relational climate underpins the entire educational process (37, 42). Furthermore, it is emphasized that this relationship must afford personalized, individualized attention to the learner (39, 43, 44).

Also important is the planning–space dimension. Martínez Clares et al. [(36), p. 270] argue for understanding tutoring as “a systematic, planned, integrated, intentional, continuous process.” Additionally, the creation of well-designed spaces, recreational, communicative, and attuned to environmental factors such as lighting and climate, is highlighted as crucial to fostering interaction among learners and between learners and educators [(51), p. 63].

Training of the accompanier is also critical, grounded in their personal maturity and development (26, 28). Specifically, Vélaz de Medrano Ureta [(29), p. 217] asserts that “the mentor must receive specific training that enables them to accurately envision their role and to develop a set of competencies for its execution.”

A final noteworthy aspect is the accompanier’s prior professional experience in accompaniment (44).

#### 3.2.10 Key competencies of accompaniment (O10)

Table 11 outlines the key competencies for educational accompaniment identified in the analyzed studies.

**TABLE 11:** Key competencies of educational accompaniment (EA).

Key competencies	Relational	Communicative	Cognitive
No. of studies (%)	22 (78.6%)	17 (60.7%)	7 (25%)
Studies	1, 3, 4, 5, 10, 11, 12, 13, 14, 15, 17, 18, 19, 20, 21, 22, 23, 24, 25, 26, 27, 28	1, 3, 4, 12, 13, 15, 17, 18, 19, 20, 21 23, 24, 25, 26, 27, 28	1, 3, 4, 20, 21, 25, 27
Explanation	Trust, collaboration, motivation	Active listening, empathy, assertiveness	Knowledge of EA, understanding of educational intervention, metacognitive ability to deploy knowledge strategically
Reference authors	Herrera-Pastor et al. (39), Crespí and López (44), Herrera-Pastor et al. (48), González-Iglesias and De la Calle Maldonado (49)	Rodríguez Herrero et al. (28), Aravena-Kenigs et al. (37), Fernández-Simo and Cid Fernández (42), Crespí and López (44) , Jiménez-Jiménez et al. (51)	Vélaz de Medrano Ureta (29)

Relational competencies refer to those that enable individuals to interact effectively and constructively with others, fostering security and trust in the accompanied individual (49). Such competencies manifest through collaborative engagement—providing support and assistance across various domains of the accompanied individual’s life—while respecting their autonomy and independence (39). These relational dynamics motivate and sustain the accompanied person’s efforts toward their goals. Establishing a climate of trust between accompanier and accompanied is essential for effective EA (48).

Communicative competencies encompass the skills that enable the accompanier to communicate effectively: listening, empathy, and assertiveness. Active listening allows the accompanied individual to articulate emotions related to their lived experience and life project (28, 51). Empathy permits the accompanier to understand the accompanied person’s situation and context without impeding their autonomous progression through the process (42). Assertiveness ensures a secure environment for feedback and constructive dialogue (37).

Cognitive competencies are required to process information, learn, solve problems, and make decisions. Specifically, they include domain-specific knowledge of the context in which accompaniment occurs; the ability to apply and adapt this knowledge to diverse contexts and situations; and metacognitive skills that enable the strategic use of knowledge and ongoing self-directed learning (29).

#### 3.2.11 Research findings (O11)

Table 12 presents the principal research findings identified in EA studies.

**TABLE 12:** Research findings in educational accompaniment (EA).

Research finding	Personal development and independence	Teacher performance	Autonomy and engagement	Equal opportunities	Child development
No. of studies (%)	9 (32.1%)	6 (21.4%)	6 (21.4%)	2 (7.1%)	2 (7.1%)
Studies	6, 9, 10, 14, 17, 18, 21, 23, 25	5, 7, 8, 12, 27, 28	11, 19, 20, 22, 24, 26	3 and 13	15 and 16
Explanation	Overcoming challenges to achieve autonomy, adult integration, and training	Improvement of the educational process and performance of novice or prospective teachers	Acquisition of competencies and advancement of personal goals in a bidirectional accompanier-accompanied dynamic	Development opportunities for individuals with special needs, recognizing their uniqueness	Support for families in the integral formation and development of their children
Reference authors	Herrera-Pastor et al. (39), Fernández-Simo et al. (43)	Aravena-Kenigs et al. (37), Castro Pérez and Moya Márquez (53)	Herrera-Pastor et al. (39), Crespí and López (44), Herrera-Pastor et al. (48)	Rodríguez Herrero et al. (28), Boroel Cervantes et al. (38)	García Lara et al. (41)

#### 3.2.12 Prospective directions (O12)

Table 13 summarizes the prospective recommendations proposed to enhance EA practice.

**TABLE 13:** Suggested prospective actions for enhanced educational accompaniment (EA).

Prospect	Consolidation of EA programs	Accompanier Training	Adaptation to university context	Family–school coordination
No. of studies (%)	8 (28.5%)	5 (17.8%)	5 (17.8%)	2 (7.1%)
Studies	6, 14, 17, 18, 22, 23, 25, 26	1, 2, 3, 4, 13	12, 19, 24, 27, 28	15 and 16
Explanation	Practical programs encompassing all facets of EA	Training accompaniers in both educational and psychological domains to address expectations, individual characteristics, and personal circumstances	Tailoring EA to the specific demands of higher education contexts	Coordinating EA activities with family involvement
Reference authors	Pàmies Rovira et al. (31), Herrera-Pastor et al. (39)	Pallarès Piquer et al. (26), Rodríguez Herrero et al. (28), Vélaz de Medrano Ureta (29)	Crespí and López (44), Castro Pérez and Moya Márquez (53)	García Lara et al. (41)

Overall, the foremost prospective direction is the consolidation of EA programs (39) and the sustained coordination of all EA interventions over time (31).

## 4 Discussion

The temporal evolution of the literature (O1) reflects a growing scholarly interest in EA. It is notable that the vast majority of authors and their institutional affiliations are based in Spain, with a smaller representation from Ibero-American countries. One explanatory factor may be the explicit recognition of accompaniment within Spanish legislation; particularly in the socio-educational domain (cf. Law 44/2007 of 13 December; Art. 30.3 of the LOMLOE of 3 May 2006). Another driver of interest, especially within Christian-inspired universities, stems from the magisterium of Pope Francis on this theme (63) and from personalist writers such as López Quintás (64), a disciple of Romano Guardini. The relative paucity of Anglo-Saxon research may be attributable to the preference for related terms, such as “mentoring,” “tutoring,” or “counseling,” rather than the more encompassing concept of accompaniment. Given that the selection criteria were limited to the use of the term accompaniment, works that use this concept under other names (e.g., mentoring) do not appear. Indeed, EA is a broader and richer construct, and one may anticipate that Anglophone scholars will increasingly adopt the term accompaniment. In any event, it should be recognized that EA subsumes methodologies such as mentoring and tutoring among others.

A key contribution of the present scoping review is the identification of four distinct theoretical models (O2), succinctly summarized in Table 3. Each model corresponds to particular domains and objectives and is associated with its own methodological orientation, as discussed below.

The four models apply to the fields of education and health. Although the evidence gathered in the literature is insufficient to identify the superiority of one model over another, there are some elements that indicate that the personalist model is recommended, even in the field of health. For example, the purpose or aim of the personalist model is broader and can include the purposes or objectives of the other models (health, excellence, autonomy). Likewise, the emphasis of the personalist model on the relational nature of human beings does not hinder but rather favors the pursuit of autonomy, the development of competencies, health, and the flourishing of the person. According to the personalist model, the educational act, like the medical act, is inherently ethical and intends the good of the person in its care (65).

Despite their divergences, these models share common features, which permit the formulation of an integrative definition of EA: an intentional educational action through which one person enlightens and supports another in their integral development, facilitating their autonomy, maturity, and realization of a fulfilling life.^1^ This definition articulates three essential dimensions: (1) the nature of educational accompaniment (a purposive educational action implementable in various modalities); (2) its content or object (to illuminate and sustain another person’s development of capacities or competencies); and (3) its ultimate aim (maturity, autonomy, and a fulfilment of life). Moreover, it meets three criteria for a robust definition (66): (a) generality, capturing the concept at its most inclusive level without privileging or excluding specific applications; (b) empirical precision, reflecting how EA manifests in practice; and (c) clarity of boundaries, delineating what falls within its scope.

Regarding research methodologies (O3), qualitative methods emerge as the most frequently employed, followed by theoretical essays and, to a lesser extent, mixed methodologies. An analysis of the relationship between theoretical models and the methodologies utilized reveals a methodological alignment in accordance with the adopted model: authors working within personalist frameworks predominantly favor theoretical essays, while those aligned with socio-educational models primarily employ qualitative approaches. Meanwhile, authors drawing on competency-based and psychosocial models, though also inclined toward theoretical or qualitative methods, incorporate mixed methodologies that include quantitative components. This trend reflects the methodological preferences inherent to each paradigm’s approach to the object of study (67, 68).

With respect to the typology of accompaniment (O4), tutoring and mentoring emerge as the most extensively studied forms, examined across a variety of contexts. The existing body of research on these modalities offers significant potential for advancing the conceptual and practical development of educational accompaniment (44, 69).

In terms of educational settings (O5), the findings highlight a predominant focus on the social domain, followed by higher education and school education, with special education being the least addressed area.

Regarding the roles of the accompanier and the accompanied (O6), the teacher is the most frequently represented figure, followed by the social educator and the social worker. Other roles—such as parents, school administrators, counselors, educational accompaniment specialists, mediators, and students themselves—appear more sporadically. The findings support the notion that the role of accompanier can be effectively fulfilled within asymmetrical relationships, provided these foster the autonomy of the accompanied individual (70). As for the accompanied, students—across all educational levels and domains—constitute the most represented group, followed by individuals who are vulnerable or at risk of social exclusion, teachers, individuals with disabilities, and school-aged children.

Regarding the benefits of educational accompaniment (O7), the three most frequently cited are: (1) the promotion of the accompanied individual’s autonomy and social inclusion; (2) comprehensive education and the development of transversal competencies; and (3) employability, particularly within the context of psychosocial studies. A notable alignment is observed between the benefits attributed to educational accompaniment and those associated with educational coaching (71).

Several challenges associated with educational accompaniment (O8) have also been identified. These include the diversity and complexity of training needs, the lack of continuity and follow-up in the accompaniment process, the accompanier’s lack of motivation, and a mismatch between the needs of the accompanied and the support offered by the accompanier. Authors such as Burgess et al. (72) emphasize the importance of acknowledging that accompaniment is not always effective, and that challenges may arise due to unclear or misunderstood roles and responsibilities of both accompanier and accompanied, as well as ambiguous boundaries within the relationship.

With respect to key elements for effective accompaniment (O9), the literature highlights the primacy of the relationship between accompanier and accompanied over other factors such as training, planning, or experience. The significance of this relationship was already underscored by Rogers (57, 58).

Concerning the key competencies for effective accompaniment (O10), most of the reviewed studies point to relational and communicative competencies. Rodriguez Berrio (73) argues that relational competencies are essential for establishing trust, creating a safe environment, and fostering personal and social development. In this regard, accompaniment is understood as a process aimed primarily at “mobilizing the resources of the accompanied individual(s)” [(74), p. 42], a process that can only occur if a strong and supportive relationship is in place.

With regard to the main research findings on educational accompaniment (O11), it must be acknowledged that there are methodological limitations that hinder the generalization of results—an issue already highlighted in other systematic reviews, particularly those focused on mentoring. Nevertheless, existing evidence points to the positive impact of accompaniment on both personal and professional development (75). Specifically, accompaniment has been shown to foster personal growth and enhance the autonomy of the accompanied individual. The findings identified in this review concerning the effectiveness of educational accompaniment are consistent with studies on mentoring in healthcare contexts, where accompaniment has been associated with improvements in students’ internal locus of control and their ability to cope with stress proactively, regardless of the specific model of accompaniment employed (76, 77). Furthermore, the quality of the relationship between accompanier and accompanied has been demonstrated to exert a significant influence on student outcomes, including in healthcare settings (78).

Regarding future directions for improving educational accompaniment (O12), our findings align with those of González-Iglesias (79) and Domingo Benito et al. (80), who argue that effective accompaniment requires respect for the accompanied person’s freedom, circumstances, expectations, and personal history. Moreover, as noted by Sánchez and Dávila (81) and Rodrigo López et al. (82), appropriate accompaniment of adolescents necessitates strong coordination and collaborative efforts among stakeholders.

## 5 Conclusion

This study has conducted a scoping review of the academic literature on educational accompaniment (EA), compiling information on theoretical models as well as key findings. This provides a foundation for offering suggestions for future research.

A first conclusion is that the term “accompaniment” appears to be particularly associated with the Spanish and Latin American context. In the Anglophone sphere, specific forms of educational accompaniment, such as mentoring and tutoring, are extensively studied, but the term “accompaniment” itself is seldom used. The dissemination of “accompaniment” within Anglophone academia could be highly beneficial, both for enriching the theoretical framework available to English-speaking researchers and for enhancing the development of EA through the extensive research already conducted on mentoring and tutoring.

Secondly, four distinct models of EA have been identified (psychosocial, personalist, competence-based, and socio-educational) which together offer a comprehensive framework for understanding and comparing the diverse approaches within the field. The conceptual analysis also supports a definition of EA that can be applied across these models although the personalist model seems to be the most comprehensive and appropriate for educational accompaniment. We offer a definition, from a personalist perspective, compatible with the various models: educational accompaniment is the intentional educational act through which one person supports and enlightens another in the development of their capacities, with the aim of fostering maturity, autonomy, and a fulfilling life.

Thirdly, the reviewed studies indicate that EA, most often implemented by educators, has a positive impact on the accompanied individuals across various contexts. The relationship between accompanier and accompanied is both a necessary condition and a core competency for effective EA. Its main benefits include personal development and autonomy, integral formation, social inclusion, and employability.

Fourthly, several challenges to effective EA have been identified, including the diversity and complexity of the accompanied individual’s needs, and the necessity for continuity over time. Additionally, effective accompaniment requires proper training (especially for the accompanier), contextual adaptation, and coordination between the family and the educational institution.

Taken together, these findings suggest that EA is an emerging and significant field of study, and that further research will enhance its implementation across multiple domains, including healthcare. As future lines of research, it would be advisable to conduct comparative studies to evaluate the effectiveness of the models in clinical and educational contexts in the field of health, both in the training of healthcare professionals and in terms of the wellbeing and flourishing of patients. It is also advisable to research educational accompaniment as part of a model for the education of healthcare professionals during their university studies, including the virtues and competencies they need for their education and future professional practice (83).

Likewise, it is advisable to conduct systematic studies focused on the concept of educational accompaniment and not just on the term itself, in order to overcome the methodological limitation, we mentioned earlier. This will enable us to better relate the findings of this study, which is geographically focused on the Spanish-American sphere, to research in the Anglo-Saxon sphere that does not use the term accompaniment but does use the concept.

## Data Availability

The original contributions presented in this study are included in this article, further inquiries can be directed to the corresponding author.

## Footnotes

1 acompañamiento educativo puede definirse como una acción educativa intencional mediante la cual una persona ilumina y apoya a otra en su desarrollo integral, facilitando su autonomía, madurez y realización de una vida plena.
